# The complete chloroplast genome sequence of *Habenaria dentata* (Orchidaceae)

**DOI:** 10.1080/23802359.2022.2080016

**Published:** 2022-06-10

**Authors:** Jin Zhang, Yishan Yang, Jianmin Tang, Yajin Luo, Huqiang Lv, Shengfeng Chai

**Affiliations:** a College of Pharmacy, Guilin Medical University, Guilin, China; b Guangxi Key Laboratory of Functional Phytochemicals Research and Utilization, Guangxi Institute of Botany, Guangxi Zhuang Autonomous Region and Chinese Academy of Sciences, Guilin, China; c Yachang Orchid National Nature Reserve Management Center, Baise, China; d Xi'an Research Institute of Chinese Lacquer Under All China Federation of Supply and Marketing Cooperatives, Xi'an, China

**Keywords:** *Habenaria dentata*, complete chloroplast genome, Orchidaceae, phylogenomics

## Abstract

*Habenaria dentata* is a rare species with high ornamental value in China. In this study, we report the complete chloroplast (cp) genome of *H. dentata* using the Illumina sequencing data. The total genome of *H. dentata* is 153,682 bp in length and the GC content is 36.62%, with a pair of inverted repeats (IRs) regions of 26,339 bp each, a large single-copy (LSC) region of 83,963 bp and a small single-copy (SSC) region of 17,041 bp. The cp genome encoded 133 genes, including 87 protein-coding genes (PCG), eight rRNA genes, and 38 tRNA genes. The maximum-likelihood phylogenetic analysis based on 12 cp genomes showed that *H. dentata* was sister to *Habenaria chejuensis* and *Habenaria ciliolaris*. This work will be valuable for genetic and phylogenetic studies on *H. dentata.*

*Habenaria* is one of the largest genera in the Orchidaceae family, including about 876 species and mainly distributed in tropical and subtropical areas (Govaerts et al. 2013). According to Flora of China, 58 species of *Habenaria* were reported in China (Zhang et al. 2016). *Habenaria dentata* (Sw.) Schltr 1919 is a species belonging to the *Habenaria* genera. It grows on the forest understory or gully sides of mountain slopes at an altitude of 190–2300 m (Chen et al. 2009). *H. dentata* has high ornamental value, and its tubers can be used medicinally, with the effect of diuretic, swelling, waist strength and kidney, treating lumbago, hernia and other diseases. However, due to its slow growth, special ecological requirements, difficulty in reproduction and long-term exploitation, the species is now very rare. *Habenaria dentata* is classified as a second-level key protected plant in the ‘National List of Key Protected Wild Plants (Second Batch)’ (http://www.iplant.cn/rep/prot/Habenaria%20dentata). To better protect *H. dentata* and provide significant genomic resources in the further study of Orchidaceae, we reported its chloroplast (cp) genome first and analyzed its structural characteristics and phylogenetic relationship.

Fresh leaves of *H*. *dentata* were collected at the Yachang Orchid National Nature Reserve, Baise, Guangxi, China (24°50′55″N, 106°21′5″E), and the specimens were preserved in the herbarium of the Guangxi Institute of Botany (contact: Shengfeng Chai, e-mail: sfchai@163.com, voucher number: SFC-20201015012). The sample collection was permitted by the Yachang Orchid National Nature Reserve Management Center, and the sampling process was followed by the Regulations of the People's Republic of China on the Protection of Wild Plants. Total genomic DNA was extracted using a modified CTAB method (Doyle And Doyle 1987), followed by double-end sequencing using the Illumina HiSeq 2000 platform, and the cp genome was assembled from scratch using the GetOrganelle script (Jin et al. 2018). Finally, we obtained the complete cp genome of *H*. *dentata* and used the online annotation software Geseq (Tillich et al. 2017) and CpGAVAS (Liu et al. 2012), using *H*. *pantlingiana* (NC_026775) as the reference genome, for cp genome annotation was spliced. Circular genome maps were drawn using the online tool OGDRAW (Lohse et al. 2007), and after proofreading and correction, the whole cp genome was submitted to GenBank (NCBI accession number: OK012095).

The complete cp genome sequence of *H. dentata* was 153,682 bp in length and showed a typical quadripartite structure, consisting of the large single-copy (83,963 bp) and small single-copy (17,041 bp) separated by a pair of inverted repeat (IR) (26,339 bp). It contains 133 protein-coding genes, 38 tRNA genes, and eight rRNA genes. The overall GC content of *H. dentata* cp genome was 36.62%, and in the LSC, SSC, and IR regions were 34.15, 28.96, and 43.04%, respectively. By comparing the cp genomes of *H. dentata*, we found that *H. dentata* is very similar to the cp genomes of previously reported *Habenaria* species in terms of gene content, gene order, and GC content.

We performed a phylogenetic analysis with 12 complete cp genomes of Orchidaceae and two species of Iridaceae as outgroups. The GenBank accession numbers of the sequences used in this paper are shown in Figure 1. Sequence alignment was performed using MAFFT (Katoh and Standley 2013), and a maximum-likelihood (ML) tree in the GTRGAMMA model was constructed using RAxML 8.2.12 (Stamatakis 2014), and the phylogenetic tree showed that *H. dentata* was clustered with *Habenaria chejuensis* and *Habenaria ciliolaris* with 100% bootstrap support (Figure 1). In conclusion, the complete cp genome of *H. dentata* was decoded for the first time, which will facilitate the species identification, molecular biology, and phylogenetic studies of *H. dentata*. This cp genome will provide important clues to better understand the phylogeny and biodiversity of *Habenaria*.

**Figure 1.**
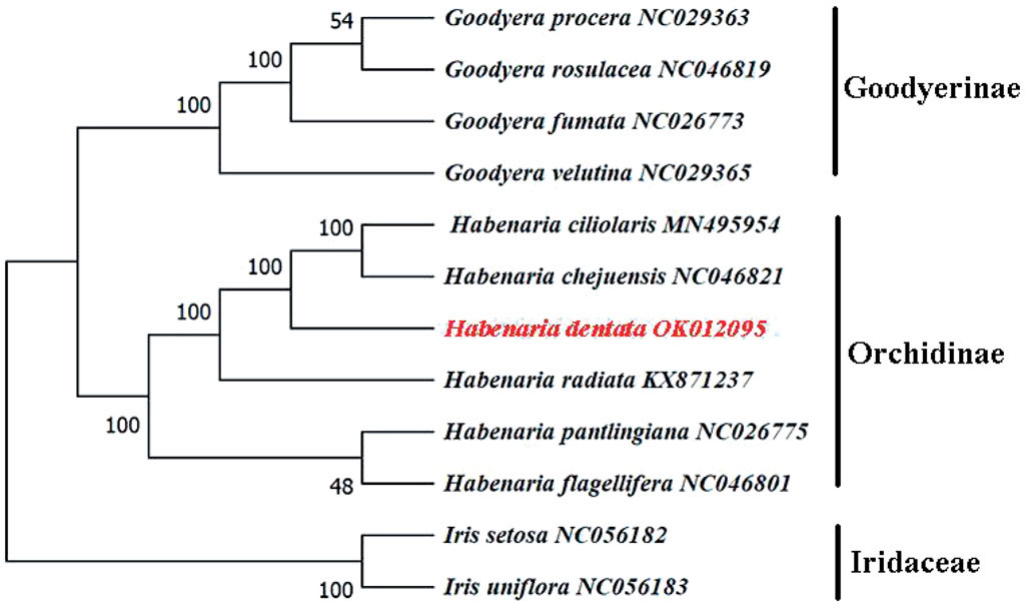
Phylogenetic tree reconstruction of 12 species based on whole chloroplast genome sequences. All sequences were downloaded from NCBI GenBank.

## Author contributions

S.C. conceived the research; S.C. collected samples; J.Z., Y.Y., J.T., Y.L., and H.L. analyzed and interpreted data; J.Z. wrote the manuscript; S.C., Y.Y., and J.Z. revised the manuscript. All authors approved the final version of the article and agreed to be accountable for all aspects of the work.

## Data Availability

The specimens of Habenaria dentata were preserved in the herbarium of the Guangxi Institute of Botany (voucher number: SFC-20201015012). The genome sequence data that support the findings of this study are openly available in GenBank of NCBI at https://www.ncbi.nlm.nih.gov/ under the accession no. OK012095. The associated BioProject, SRA, and Bio-Sample numbers are PRJNA759395, SRR15685573, and SAMN21166999, respectively. Tree file of 12 species and genes for phylogenetic analysis were deposited at Figshare: https://doi.org/10.6084/m9.figshare.16910296.
